# An Interpretable Kolmogorov–Arnold Network for FTIR Detection and Quantification of Adulteration Across Diverse Food Matrices

**DOI:** 10.3390/foods15172949

**Published:** 2026-08-22

**Authors:** Abdulhamid Batayhi, Muhammed Özgölet, Osman Sagdic

**Affiliations:** 1 Department of Mechatronics Engineering, Faculty of Mechanical Engineering, Yildiz Technical University, 34220 Istanbul, Türkiye; abdulhamid.batayhi@std.yildiz.edu.tr; 2 Department of Food Engineering, Faculty of Chemical and Metallurgical Engineering, Yildiz Technical University, 34220 Istanbul, Türkiye; osagdic@yildiz.edu.tr

**Keywords:** food adulteration, ATR-FTIR, olive oil, interpretable machine learning, chemometrics, food authentication

## Abstract

Economically motivated adulteration of olive oil, coffee and fruit juice is a persistent food-fraud problem for which Fourier-transform infrared (FTIR) spectroscopy with chemometrics offers rapid screening. Linear partial least squares (PLS) is interpretable but cannot capture non-linear mixing; neural networks add flexibility at the cost of becoming black boxes. We evaluated a Kolmogorov–Arnold network (KAN), which places learnable univariate functions on its edges and is therefore intrinsically interpretable, against PLS, support-vector regression, random forests, a multilayer perceptron and a one-dimensional convolutional network on three attenuated total reflectance (ATR)–FTIR datasets (olive oil + sunflower oil, coffee + malt flour, orange juice + apple juice; approximately 350, 400 and 400 spectra). All models were compared under identical, leakage-free validation that splits spectra by physical sample. The compact KAN was consistently competitive (cross-validated coefficients of determination (R^2^) = 0.86, 0.93 and 0.69) and yielded closed-form equations whose variables map to recognised vibrational bands and whose importance ranking agrees with SHapley Additive exPlanations (SHAP; Spearman ρ = 0.86–0.90); symbolic conversion costs no accuracy. We also report the following limits: PLS was strongest where the chemistry was linear (coffee) and the multilayer perceptron was strongest on fruit juice, whose equation is the weakest (R^2^ = 0.47–0.75 across seeds); a parameter-matched perceptron matched the KAN’s accuracy; and leave-one-brand-out validation degraded every model. The KAN is therefore a promising, compact and genuinely transparent alternative under controlled multi-matrix conditions, not a deployment-ready method.

## 1. Introduction

Economically motivated food adulteration—the partial replacement of a valuable product with a cheaper one—erodes consumer trust and can threaten health. Olive oil is among the most frequently adulterated commodities, commonly extended with cheaper seed oils such as sunflower oil; roasted coffee is diluted with grains and other plant materials; and fruit juices are stretched with cheaper juices or added sugars [1]. Reliable, rapid, and low-cost screening methods are therefore central to food quality control. Vibrational spectroscopy, and in particular Fourier-transform infrared (FTIR) spectroscopy in the attenuated-total-reflectance (ATR) mode, provides a fast, reagent-free fingerprint of a sample’s chemical composition and has become a workhorse for authenticity testing across edible oils, beverages and other matrices [2,3]. Converting that fingerprint into a quantitative answer, however, depends on the calibration model applied to the spectra.

The dominant calibration approach is partial least squares (PLS) regression [4], usually preceded by scatter and baseline correction such as the standard normal variate transformation [5] and Savitzky–Golay smoothing or differentiation [6]. PLS is valued precisely because it is interpretable: its regression coefficients can be read back onto wavenumbers and related to chemical bands. This transparency is important in food analysis, where a method must be defensible to regulators and not merely accurate. PLS is, however, a linear model, and when the relationship between spectrum and composition departs from linearity it can be outperformed by more flexible learners such as support-vector regression [7], random forests [8] and, increasingly, deep neural networks, including one-dimensional convolutional networks for spectral calibration [9]. The cost of that flexibility is interpretability: such models are effectively black boxes. Post hoc explainers such as SHAP [10] can attribute a prediction to input features, but they do not expose the model’s functional form, and the explanation is an approximation generated after the fact rather than the model itself.

The Kolmogorov–Arnold network (KAN) targets exactly this trade-off [11]. While a conventional network applies fixed non-linear activations at its nodes and learns linear weights on its edges, a KAN learns a univariate function on every edge and sums them at the nodes. Because each connection carries an explicit, inspectable function, a trained KAN can be pruned and converted into a compact symbolic formula, an approach that has been used to recover physical relationships from data [12]. This makes the KAN conceptually well suited to spectroscopy, where the quantity of interest is built up from contributions of individual spectral regions: each learned edge function behaves as a non-linear per-feature response, and the final model can be written down as an equation.

Kolmogorov–Arnold networks have begun to appear in spectroscopic analysis over the last two years. Radial-basis-function variants have been applied to near-infrared prediction of coal calorific value [13], and KAN modules have been combined with convolutional and recurrent backbones for surface-enhanced Raman cancer screening [14], Raman diagnosis of membranous glomerulonephritis [15] and laser-tweezer Raman identification of bacterial spores [16]. In these studies, the KAN functions chiefly as a predictive component—often hybridised with another architecture, or with the B-spline bases replaced by radial basis functions—and no closed-form equation is extracted or chemically interpreted. To our knowledge the architecture has not been evaluated for mid-infrared food-authenticity analysis, and its intrinsic interpretability has not been exploited in that setting.

Existing FTIR food-authentication models face a structural trade-off. Linear calibration models such as PLS are interpretable precisely because their regression coefficients map back onto wavenumbers and hence onto vibrational bands, but they cannot represent non-linear spectrum–composition relationships. The flexible alternatives—support-vector regression, random forests, multilayer perceptrons, and one-dimensional convolutional networks—recover that flexibility but expose no functional form, so any explanation must be reconstructed after the fact by an external attributor such as SHapley Additive exPlanations (SHAP) [10]. The element missing from this literature is therefore not the KAN as such: it is a calibration model that is simultaneously non-linear and intrinsically interpretable, evaluated across chemically distinct food matrices under a validation protocol that does not leak replicate scans between training and test partitions. The Kolmogorov–Arnold network is not introduced here; it was proposed by Liu et al. [11]. Our contribution is its first systematic evaluation in this setting: a benchmark against five established calibration models under one leakage-free protocol, on three newly collected and chemically distinct matrices, together with the extraction, stability assessment and chemical interpretation of the resulting symbolic equations.

Here, we assess whether an intrinsically interpretable KAN can serve as a practical calibration model for FTIR food-authenticity screening across three chemically distinct matrices: olive oil adulterated with sunflower oil (a lipid-in-lipid system), roasted coffee adulterated with malt flour (a roasted carbohydrate matrix), and orange juice adulterated with apple juice (an aqueous sugar–acid system). Olive oil, coffee and fruit juice are among the most economically valuable food commodities worldwide and are frequently subjected to economically motivated adulteration because of their high market demand, price differences and complex supply chains [17,18,19]. These matrices span a deliberate range of difficulty and chemistry, allowing us to test the method where calibration is near-linear and where it is not. We benchmark the KAN against five established models under a single, strictly leakage-free validation protocol; we report quantification accuracy, adulteration-detection performance, and limits of detection; and we examine interpretability directly by extracting the KAN’s symbolic equations, mapping their variables to vibrational bands, and comparing the network’s intrinsic feature importance with post hoc SHAP attribution. The three matrices were newly collected for this study. Our aim is not to claim that the KAN is the most accurate possible model, but to establish, honestly, whether transparency can be obtained without sacrificing competitiveness.

## 2. Materials and Methods

### 2.1. Samples and FTIR Spectral Acquisition

The five commercial brands of each product were purchased from local supermarkets in Istanbul, Türkiye, during February, March/2026; all products were within their labelled shelf life at the time of measurement. Olive oil samples were sourced from different geographical regions of Türkiye. Two brands were sourced from the Northern Aegean region, specifically from Ayvalık and Edremit (Balıkesir), while one brand originated from the Southern Aegean region (Muğla and Akhisar). One brand was sourced from Tarsus (Mersin), representing the Mediterranean region, whereas the fifth brand consisted of olive oils sourced from different regions of Türkiye. Coffee samples consisted of medium-roasted coffee obtained from *Coffea arabica*. Five commercial brands were included, three of which were imported from Brazil, while the country of origin was not specified on the product labels of the remaining two brands. Commercial 100% orange juices were obtained from local supermarkets in Türkiye. The selected products represented commonly consumed orange juice products available in the Turkish market and contained no added ingredients or additives, according to their product labels. To simulate economically motivated adulteration, olive oil samples were adulterated with sunflower oil, orange juice samples with apple juice, and ground coffee samples with malt flour procured from local supermarkets in Istanbul, as these adulterants are among the commonly reported substitutes for the respective products. Blends were prepared gravimetrically for the adulterated samples using an analytical balance, homogenised by a vortex mixer for 1 min, and then prepared and measured at 20 ± 2 °C.

For olive oil, adulterated samples were prepared at ten concentration levels ranging from 3% to 30% (*v*/*v*) with 3% increments. For orange juice and ground coffee, adulteration levels ranged from 5% to 50% (*w*/*w*) with 5% increments. Authentic (0%) samples were also included in the dataset. Each adulterated mixture was prepared by thoroughly homogenizing the authentic product with the corresponding adulterant before spectral acquisition. For each adulteration level, five independent FTIR spectral acquisitions were collected after repositioning the sample on the ATR crystal to account for instrumental and sampling variability. A larger number of replicate spectra (20 spectra per authentic olive oil sample and 30 spectra per authentic fruit juice and coffee sample) was intentionally collected to better characterize the spectral variability of authentic samples and to achieve a more balanced dataset for machine learning analysis. All spectra were recorded on an FTIR spectrometer (Bruker Tensor 27, Bruker Optics GmbH & Co. KG, Ettlingen, Germany) equipped with an ATR accessory, at a resolution of 4 cm^−1^ over the infrared range of 4000–600 cm^−1^, with 16 scans per spectrum.

### 2.2. Datasets and Group Structure

The three datasets are summarised in Table 1. The olive oil dataset comprised approximately 350 spectra, with sunflower oil at 0–30% in 3% steps (authentic olive oil 70–100%); the coffee and fruit juice datasets each comprised approximately 400 spectra, with malt flour and apple juice, respectively, at 0–50% in 5% steps (authentic fraction 50–100%). Each dataset was prepared from 55 physical samples: five brands of the authentic product, each blended at one authentic and ten adulteration levels. Every physical sample (one prepared blend) was scanned several times, with five replicate acquisitions per blend and additional scans for the authentic samples, as described above. Because replicate scans of the same physical sample are far more similar to one another than to scans of other samples, treating individual scans as independent observations would leak information between training and test sets and inflate apparent performance. We therefore recorded, for every spectrum, the identity of its physical sample and used it as the grouping variable throughout (Section 2.5). The regression target was the authentic-fraction percentage; because the two components sum to 100%, the root-mean-square error, R^2^, mean absolute error and ratio of performance to deviation are numerically identical whether expressed for the authentic or the adulterant fraction. A binary adulteration label (adulterated if the authentic fraction was below 100%) was derived from the composition for the detection task.

Five brands do not characterise the full spectral variability of these commodities, and we do not claim otherwise; Section 3.5 quantifies how far the calibrations transfer to a brand that was not seen in training. What the design does support is the question this study asks. Because the physical sample is the splitting unit, every reported performance estimate requires a model to generalise to blends it has never seen rather than to interpolate between correlated replicate scans of a blend it has. Within that scope the comparison between the six calibration models is controlled and fair; outside it—across brands, origins, seasons, batches and instruments—it is not yet established.

### 2.3. Spectral Preprocessing

Preprocessing was selected per matrix on the training data only, using a PLS proxy under the same grouped cross-validation as used for model selection, to avoid any information leakage. The candidates were the standard normal variate transformation (SNV) [5], a first-derivative Savitzky–Golay filter (SG1) [6], their combination (SNV + SG1), and multiplicative scatter correction (MSC). SNV was selected for olive oil and coffee. For fruit juice, whose spectra are dominated by a strong, sloping water background, SNV alone left the calibration unstable and SG1 was selected instead; this is consistent with the established role of derivative preprocessing in suppressing baseline effects in aqueous matrices. Section 3.6 reports an explicit preprocessing ablation that confirms each of these selections.

### 2.4. Calibration Models

**Figure 1:**
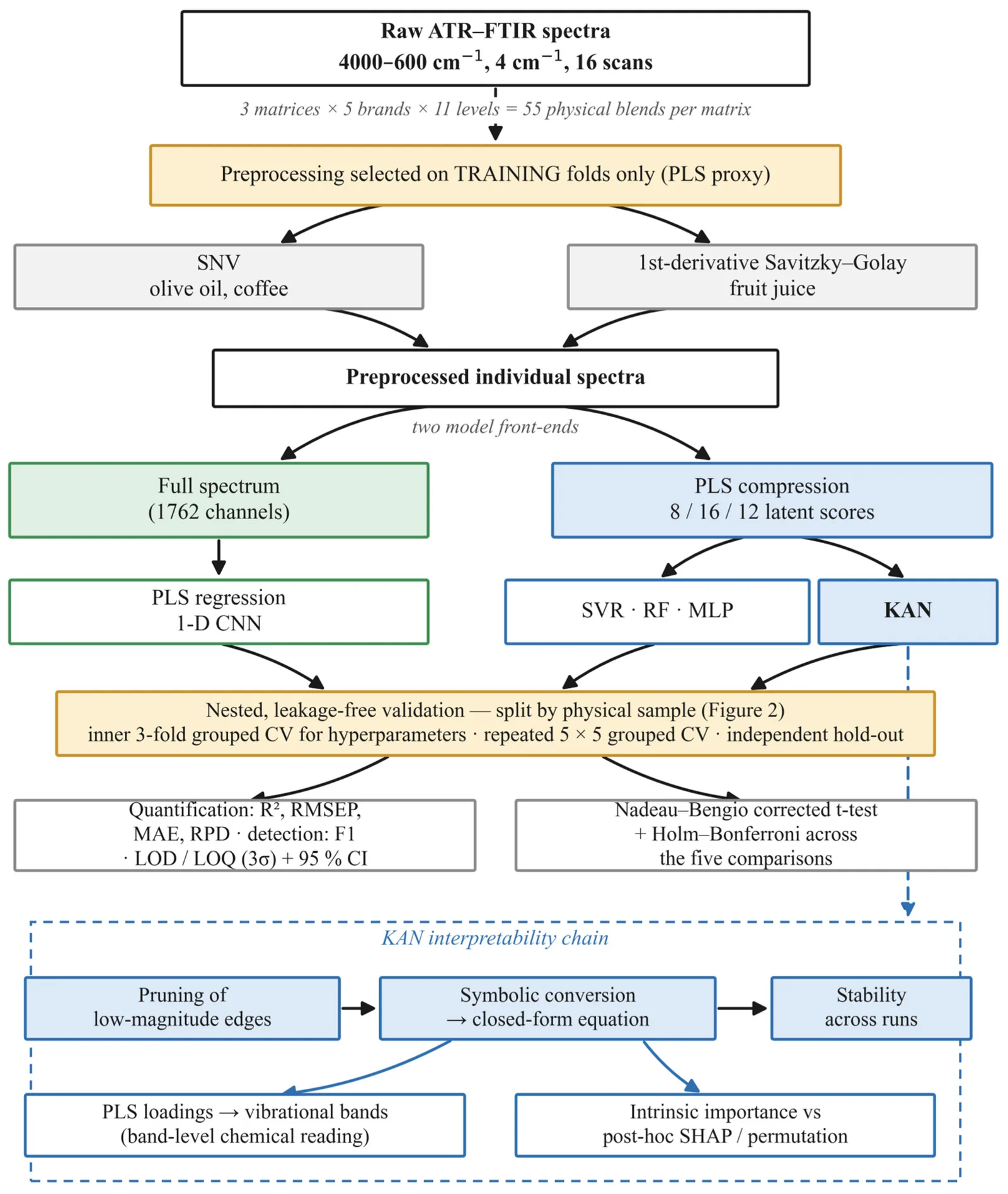
Analysis pipeline. Preprocessing is selected on training folds only; PLS regression and the igure 2 1D CNN consume the full preprocessed spectrum, while SVR, the random forest, the MLP, and the KAN consume PLS latent scores. The dashed panel shows the KAN interpretability chain.

Six models were compared. PLS regression [4] and a one-dimensional convolutional neural network [9] were applied to the full preprocessed spectrum (1762 channels). Support-vector regression with a radial-basis-function kernel [7], a random forest [8], a multilayer perceptron and the KAN [11] were applied to PLS latent scores; the number of retained scores was treated as a per-model hyperparameter, and the values selected by the inner cross-validation for the KAN were 8, 16 and 12 for olive oil, coffee and fruit juice, respectively. The KAN therefore never receives a spectrum directly: it operates on the PLS latent scores, which is why its symbolic equation is written in the latent variables x_1_…x_8_ and must be mapped back to wavenumbers through the PLS loadings (Section 3.8). Compressing the spectrum to a small set of decorrelated, supervised PLS scores before the KAN both stabilises training and keeps the resulting equation compact and interpretable. The KAN used a single hidden layer of width three with cubic B-spline edge functions; inputs were min–max scaled into the spline grid. A broader width search was evaluated but did not improve cross-validated accuracy at the present sample sizes; therefore, the compact configuration, which is also the one from which the equation is read, was retained. The complete pipeline is summarised in Figure 1.

The KAN was trained with Adam (learning rate 5 × 10^−3^, batch size 128, 300 steps; 250 steps for the equation model, with 60 further steps after pruning). A single regularisation weight, λ = 1 × 10^−3^, multiplies a composite penalty on the learned activations that combines an L1 term (relative weight 1.0) with an entropy term (relative weight 2.0), the latter encouraging sparse, well-separated edge functions. No penalty was applied directly to the B-spline coefficients, and no weight decay was used. After training, the network was pruned at node and edge magnitude thresholds of 1 × 10^−2^ and 3 × 10^−2^ before symbolic conversion. Capacity was additionally controlled by the PLS compression and by the narrow hidden layer (width 3); these are complementary to the explicit penalty rather than a substitute for it. Section 3.6 reports an ablation over λ, the spline grid size, the spline order, and the hidden width, and shows that λ has a narrow usable range.

### 2.5. Leakage-Free Validation

**Figure 2:**
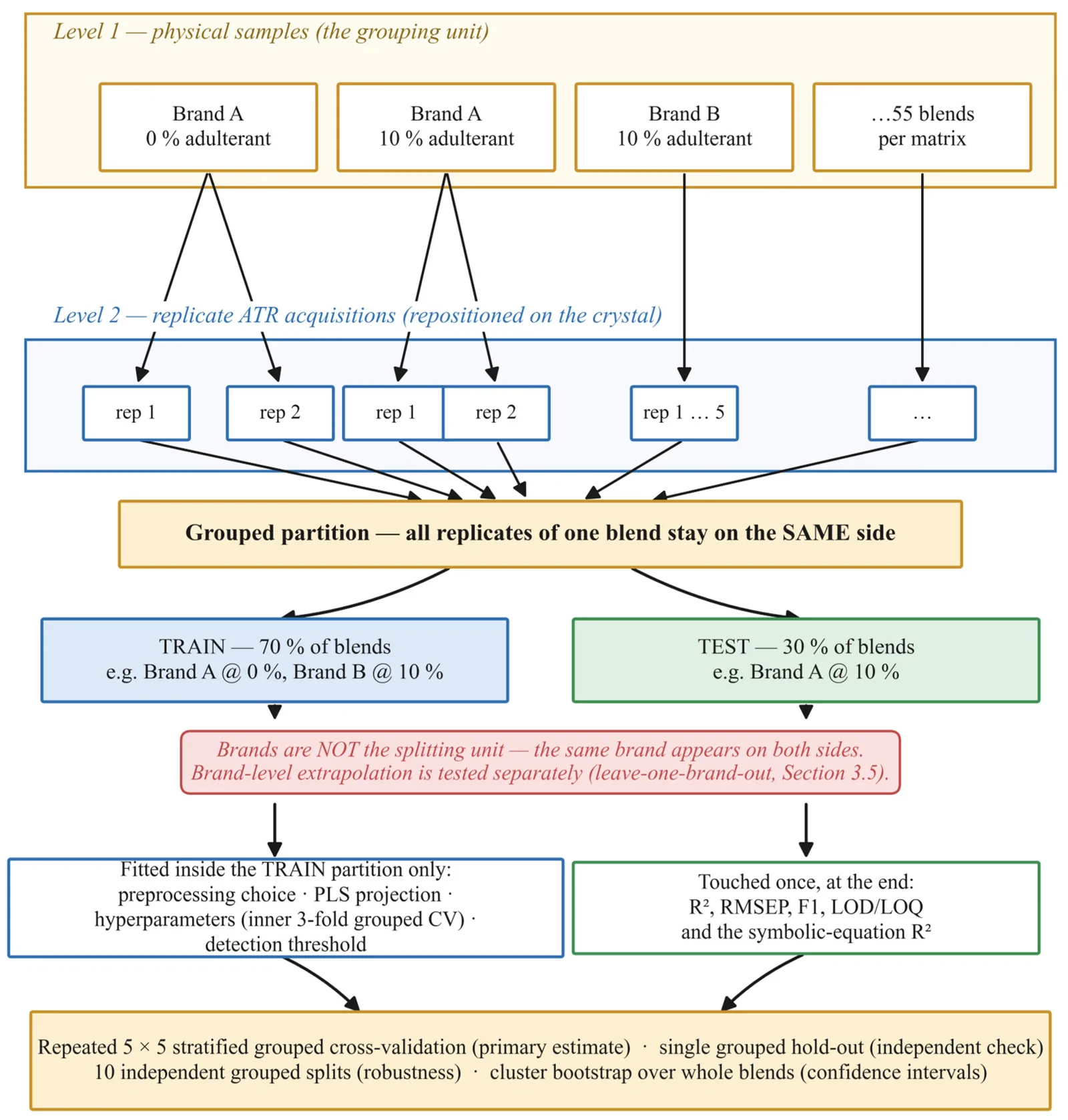
Leakage-free splitting protocol. Replicate acquisitions are nested within physical blends, and blends—not brands and not individual spectra—are the unit that is partitioned. Preprocessing, PLS projection, hyperparameters, and the detection threshold are fitted inside the training partition only.

The unit of splitting is the physical sample, defined as one prepared blend—one brand at one adulteration level—of which each matrix contains exactly 55 (5 brands × 11 levels). Every replicate scan of a blend was assigned to the same partition, so no blend contributed spectra to both the training and test sets. The protocol is illustrated in Figure 2. We emphasise that the brand is not the splitting unit: the same brand appears in both partitions at different adulteration levels, and brand-level extrapolation is assessed separately in Section 3.5.

The primary estimate was repeated using stratified group k-fold cross-validation (five repeats of five folds), with preprocessing, PLS compression, hyperparameter selection, and the detection threshold all determined inside the training folds only. Hyperparameters were chosen by an inner three-fold grouped cross-validation on the training partition, with an equal, light tuning budget for every model, so that no model was favoured by a larger search. As an independent check, we also report a single grouped hold-out (approximately 70%/30% of samples) and, to confirm that conclusions do not depend on any one split, the distribution of hold-out performance over ten independent grouped splits.

### 2.6. Performance Metrics and Statistical Analysis

Quantification was assessed by the coefficient of determination (R^2^), the root-mean-square error of prediction (RMSEP), the mean absolute error and the ratio of performance to deviation (RPD). Adulteration detection was assessed by the F1 score and accuracy at a threshold chosen on the training folds. The limit of detection was estimated following the 3σ convention (LOD = 3.3 σ/S, where σ is the residual standard deviation and S the calibration slope), with the limit of quantification at 10 σ/S. Ninety-five-percent confidence intervals were obtained by a cluster bootstrap that resamples whole physical samples rather than individual scans, so that the intervals reflect the replicate structure of the data rather than treating correlated scans as independent. Models were compared on the per-fold scores using the Nadeau–Bengio corrected resampled *t*-test, which inflates the variance of the paired differences to account for the overlap between cross-validation training sets, and the resulting *p*-values were adjusted across the five comparisons using the Holm–Bonferroni procedure. This is a deliberately conservative protocol: an uncorrected paired *t*-test on overlapping folds would report more differences as significant than the data support.

The limit of detection (LOD) and the limit of quantification (LOQ) are reported with ninety-five-percent confidence intervals, obtained using the same cluster bootstrap used elsewhere in the paper, resampling whole physical samples rather than individual scans (2000 resamples).

### 2.7. Interpretability Analysis

For each food, the compact KAN was pruned and converted to a closed-form symbolic equation in its PLS scores, and the equation’s accuracy on the hold-out set was compared with that of the underlying spline network. The learned edge functions were plotted against the corresponding linear (PLS) effects to visualise where the model used non-linearity, and the PLS loadings of the equation’s variables were overlaid on the FTIR spectrum to relate them to vibrational bands [2,20]. Finally, the KAN’s intrinsic feature importance (the magnitude of each learned edge function) was compared with two post hoc importance measures—model-agnostic permutation importance and SHAP [10]—by Spearman rank correlation.

### 2.8. Software

Analyses were performed in Python 3.13 using scikit-learn 1.8 [21] for the partial least squares, support-vector regression, random forest, and preprocessing routines; PyTorch 2.10 [22] for the multilayer perceptron and the one-dimensional convolutional network; and the pykan library (version 0.2.8) for the Kolmogorov–Arnold network. NumPy 2.4.6 and SciPy 1.17.1 were used for numerical computation and the SHAP 0.52.0 package [10] for post hoc attribution. The complete analysis pipeline and the raw spectra are openly available (see the Data Availability Statement), and all results in this paper can be reproduced from them with fixed random seeds.

## 3. Results and Discussion

### 3.1. Spectral Characteristics and Chemistry

**Figure 3:**
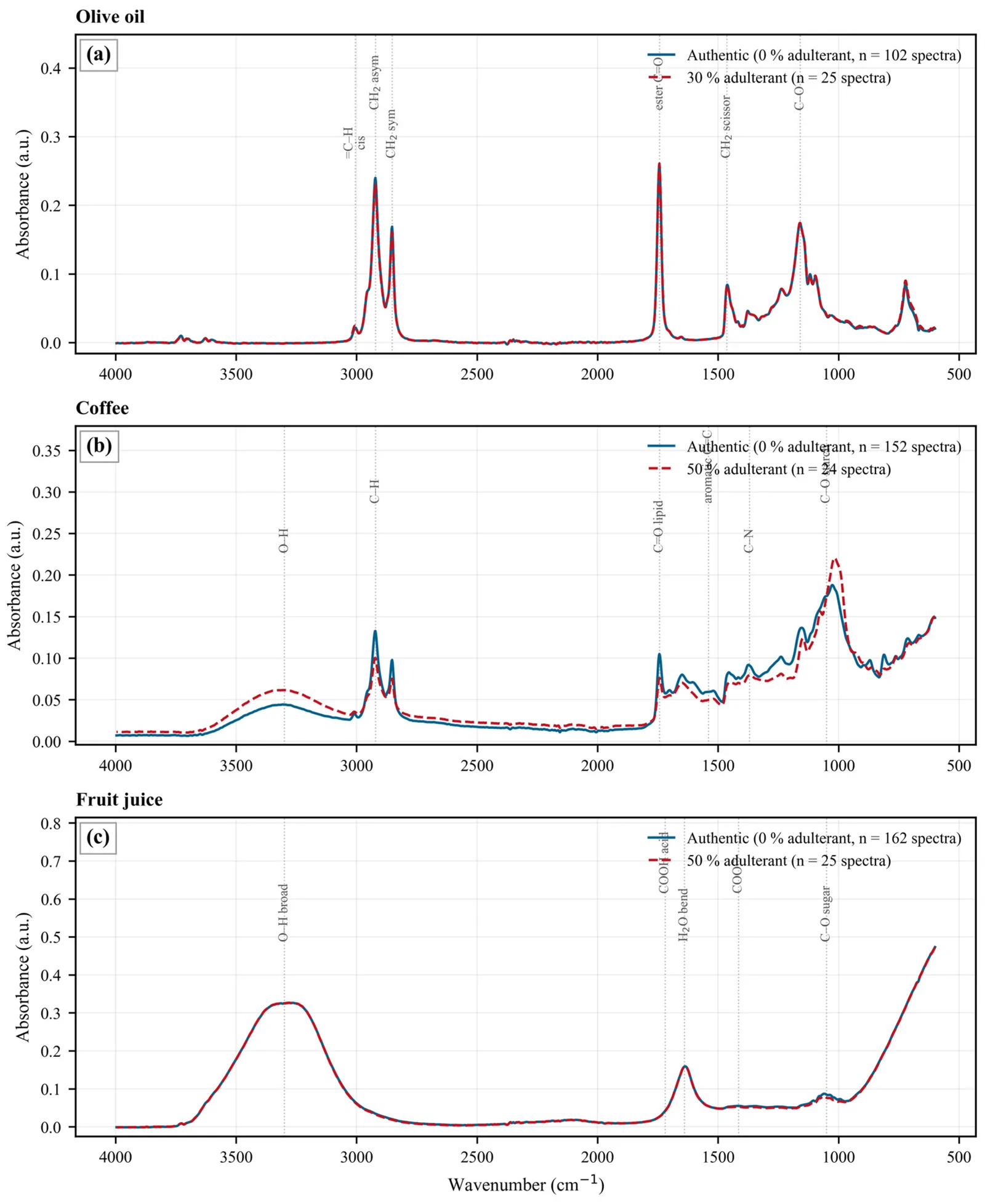
Mean raw ATR–FTIR spectra of the authentic product versus the most heavily adulterated blend for (**a**) olive oil, (**b**) coffee and (**c**) fruit juice, with diagnostic wavenumbers annotated. Spectra were averaged over all replicate acquisitions and are shown before preprocessing; the number of contributing spectra is given in each legend. Models were trained on individual preprocessed spectra, not on these averages.

The three matrices presented markedly different FTIR fingerprints (Figure 3). Figure 3 shows mean raw spectra—averaged over all replicate acquisitions of the authentic samples and of the most heavily adulterated blends, respectively, before any preprocessing—with the number of contributing spectra given in each legend. These averages are used for display and band assignment only: all models were trained and evaluated on individual spectra after per-matrix preprocessing was fitted within each training fold, and the KAN, as described in Section 2.4, operates on PLS latent scores rather than on spectra.

The olive oil spectra (Figure 3a) were dominated by the triglyceride signature of edible oils: =C–H stretching of cis-unsaturated fatty acids near 3006 cm^−1^, asymmetric and symmetric CH_2_ stretching of the acyl chains at 2922 and 2853 cm^−1^, the ester carbonyl (C=O) stretch at 1743 cm^−1^, CH_2_ scissoring at 1464 cm^−1^, and the C–O stretching envelope near 1160 cm^−1^ [2,20]. Sunflower oil differs from olive oil chiefly in its higher linoleic acid content, which perturbs the 3006 cm^−1^ and 1654 cm^−1^ regions and the relative intensities within the fingerprint region. Critically, these are intensity effects on shared bands rather than new bands: the mean spectra of pure olive oil and of the 30% sunflower blend are almost superimposed, which is why this is the most demanding of the three calibrations.

The coffee spectra (Figure 3b) showed a broad O–H envelope near 3300 cm^−1^, lipid C–H stretches at 2922 and 2853 cm^−1^, the lipid ester carbonyl at 1743 cm^−1^, aromatic C=C and melanoidin features near 1540 cm^−1^ associated with Maillard products, and a strong carbohydrate C–O stretching band at 1020–1080 cm^−1^. That band is the principal discriminant: malt flour introduces a pronounced starch C–O feature that pure roasted coffee lacks, and the difference is clearly visible between the two mean spectra. This is consistent with the near-linear, high-accuracy calibration obtained for this matrix.

The fruit juice spectra (Figure 3c) were governed by the O–H stretch near 3300 cm^−1^ and the water bending mode near 1640 cm^−1^, with the carboxylic C=O of citric and malic acid near 1720 cm^−1^ and the corresponding COO^−^ feature near 1415 cm^−1^. The sugar information that discriminates orange from apple juice is confined to the weak C–O region at 1000–1100 cm^−1^ and is superimposed on a dominant, sloping aqueous background. The authentic and 50% adulterated mean spectra are visually almost indistinguishable, which explains both the selection of first-derivative preprocessing for this matrix and the high limits of detection reported for it in Section 3.3.

### 3.2. Quantification Benchmark

**Figure 4:**
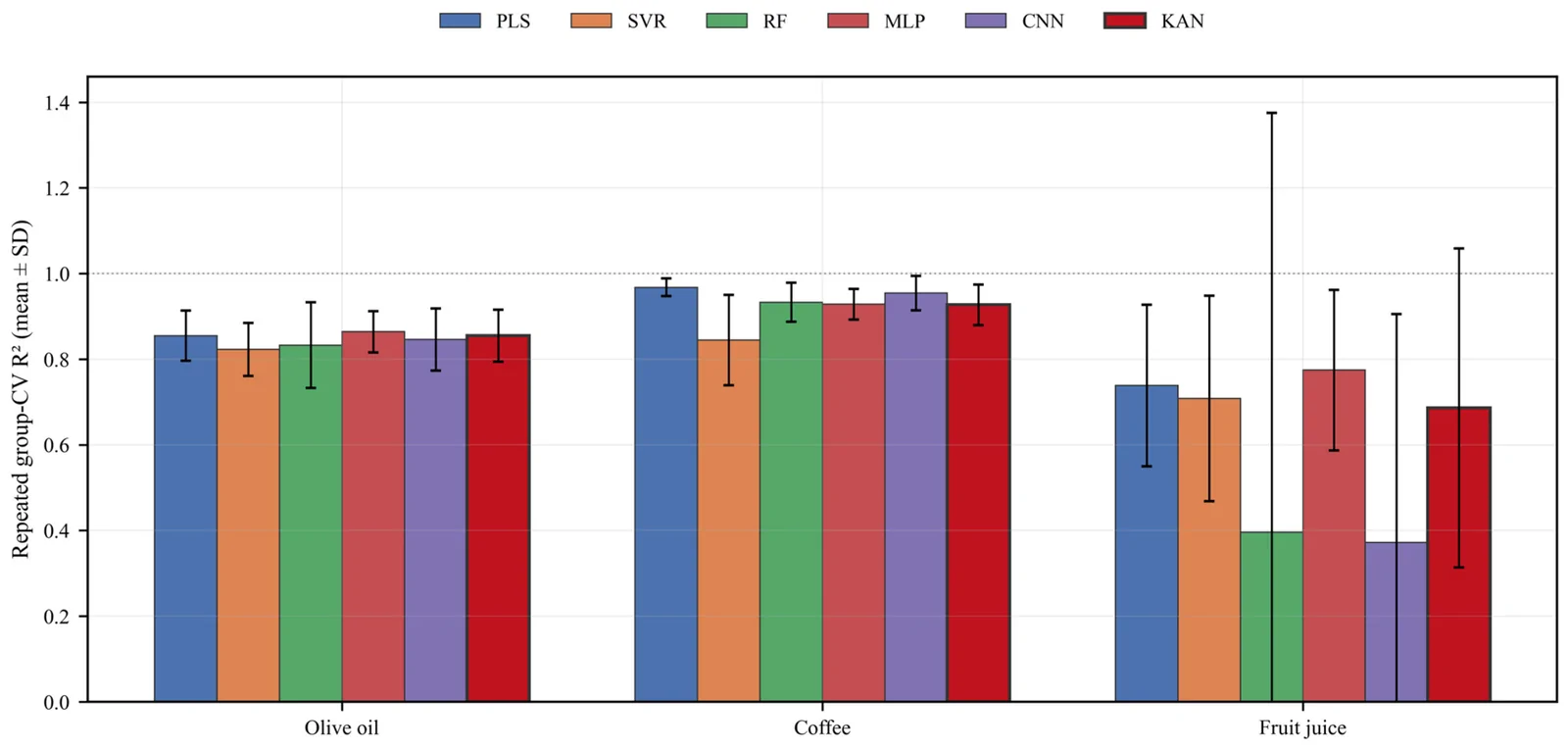
Repeated (5 × 5) group-cross-validated R^2^ by model for each food matrix (mean ± standard deviation across folds); the Kolmogorov–Arnold network (KAN) is outlined. The dotted line marks R^2^ = 1; error bars extending above it reflect the fold-to-fold spread of the random forest and convolutional models on fruit juice.

Cross-validated quantification accuracy is reported in Table 2 and summarised in Figure 4. A clear difficulty gradient emerged across the three chemistries. For coffee, all models performed well and the linear PLS model was best (R^2^ = 0.968); the KAN was competitive (R^2^ = 0.927) but significantly below PLS (Holm-corrected *p* = 0.007), the expected outcome for a near-linear calibration where added model flexibility cannot improve on an already near-optimal linear fit, and we report it as such. For olive oil—the chemically subtle lipid-in-lipid case—the KAN (R^2^ = 0.855) was statistically indistinguishable from every baseline except support-vector regression, matching both PLS (R^2^ = 0.855; corrected *p* = 0.99) and the numerically strongest model, the multilayer perceptron (R^2^ = 0.864; *p* = 1.0), and significantly exceeding only support-vector regression (*p* = 0.026). For fruit juice—the hardest, aqueous matrix—the multilayer perceptron was numerically strongest (R^2^ = 0.774); the KAN (R^2^ = 0.686) was statistically indistinguishable from PLS, support-vector regression, the random forest and the multilayer perceptron, and significantly outperformed the convolutional network (*p* = 0.007), which collapsed on this aqueous data (R^2^ = 0.37, with large fold-to-fold variance). The random forest likewise collapsed in mean accuracy (R^2^ = 0.40) but with variance so large that its difference from the KAN did not survive correction. Across all three foods, the KAN achieved this competitiveness with 580–1060 trainable parameters, fewer than the tuned baselines. We are careful not to read that as an efficiency advantage: the baselines were tuned for accuracy rather than for size, and a multilayer perceptron matched to the KAN’s parameter count performs equally well (Section 3.6).

**Figure 5:**
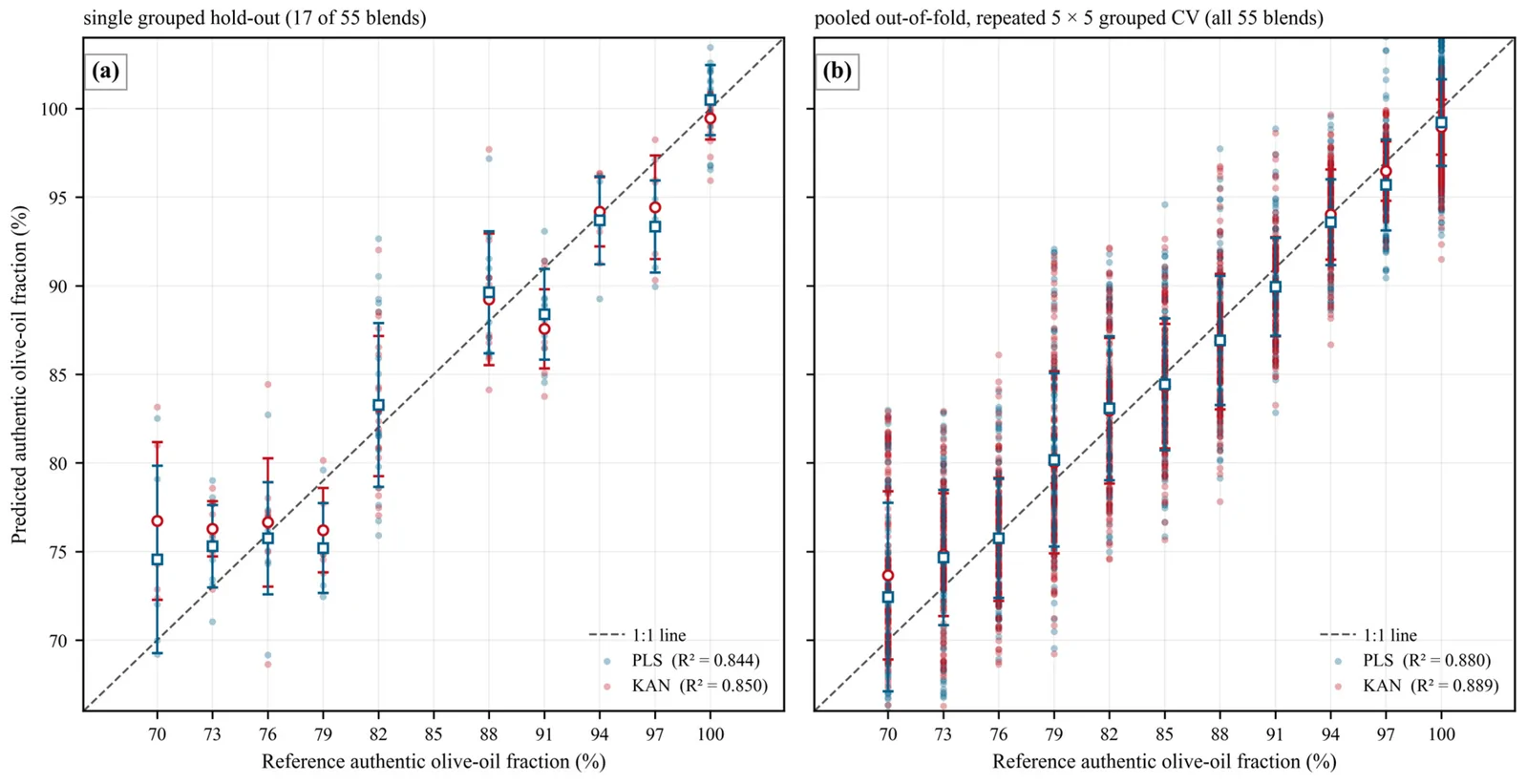
Predicted versus reference authentic olive oil fraction. (**a**) Independent grouped hold-out (17 of the 55 physical samples), comparing PLS and the KAN. The 85% level is absent from this particular partition because all five of its blends fell on the training side, and the 70% level is represented by a single blend (five replicate scans), which is why the scatter there is larger and less well-determined than elsewhere. (**b**) The same comparison built from pooled out-of-fold predictions over the repeated 5 × 5 grouped cross-validation, in which every one of the 55 blends and all eleven concentration levels appear, each predicted only by models that never saw it; open markers give the level-wise mean and bars the level-wise standard deviation. The R^2^ in panel (**b**) is computed on pooled out-of-fold predictions across the whole dataset and is therefore not the same quantity as the mean per-fold R^2^ of Table 2. The red circles and blue squares represent the mean predicted values at each reference concentration level for the KAN and PLS models, respectively. The error bars represent the standard deviation.

On the independent grouped hold-out, the same ordering held, with the KAN giving the lowest RMSEP for olive oil (3.8%); the olive oil hold-out parity of PLS and the KAN is shown in Figure 5a, and Figure 5b shows the same comparison pooled over the repeated cross-validation. Overall, the KAN was never the weakest model and was tied-best on the two harder matrices, while remaining the most compact of the learners tested—a property we do not present as an advantage in itself, for the reason given in Section 3.6.

These outcomes are matrix-specific and should be read as such. For coffee, the linear PLS calibration is the best model (R^2^ = 0.968 against 0.927 for the KAN; Holm-corrected *p* = 0.007). This is the signature of a near-linear spectrum–composition relationship, in which additional model flexibility has nothing to contribute, and it is the expected result rather than a shortfall of the KAN; the leave-one-brand-out results of Section 3.5 reinforce it, PLS being also the most stable model across unseen brands on this matrix (R^2^ = 0.976 ± 0.006). For fruit juice, the multilayer perceptron is numerically strongest (R^2^ = 0.774 against 0.686), although the difference does not survive correction for multiple comparisons; the KAN’s contribution on this matrix is a compact, interpretable alternative rather than higher accuracy, and—as Section 3.7 shows—the fruit juice equation is also the least reliable of the three, so the interpretability argument is weakest precisely where the chemistry is hardest. For olive oil, the chemically subtle lipid-in-lipid case, no model separates from the others except support-vector regression, so the matrix discriminates between calibration strategies least of the three.

### 3.3. Adulteration Detection and Limit of Detection

Binary detection was strong, but for the grosser adulteration levels, it was close to saturation: F1 scores exceeded 0.9 for olive oil and coffee, reflecting that large adulterant fractions are trivially separable. The more informative quantity is the limit of detection (Table 3). For olive oil, the estimated detection limits were closely similar across all six models (13.7–14.9% sunflower oil) and their confidence intervals overlap almost completely, so no model can be distinguished from another on this criterion; the 0.1 percentage-point difference between the KAN and PLS carries no statistical weight. For coffee, the limits ranged from 8.2% (convolutional network) to 14.9% (support-vector regression), with the KAN at 10.3%; here, the intervals separate the best models from support-vector regression but not from each other. For fruit juice, the limits were high for every model (30.7–55.5% apple juice) and the intervals overlap throughout—the KAN’s nominally worst value of 55.5% has an interval of 22.7–79.7%, which spans every other model—so the correct statement is that no model achieves useful trace detection on this matrix rather than that any one model is worse. FTIR quantification of apple juice in orange juice is therefore a screening-level rather than a trace-detection capability, and this is a limitation of the matrix rather than of any single calibration model.

### 3.4. Robustness

**Figure 6:**
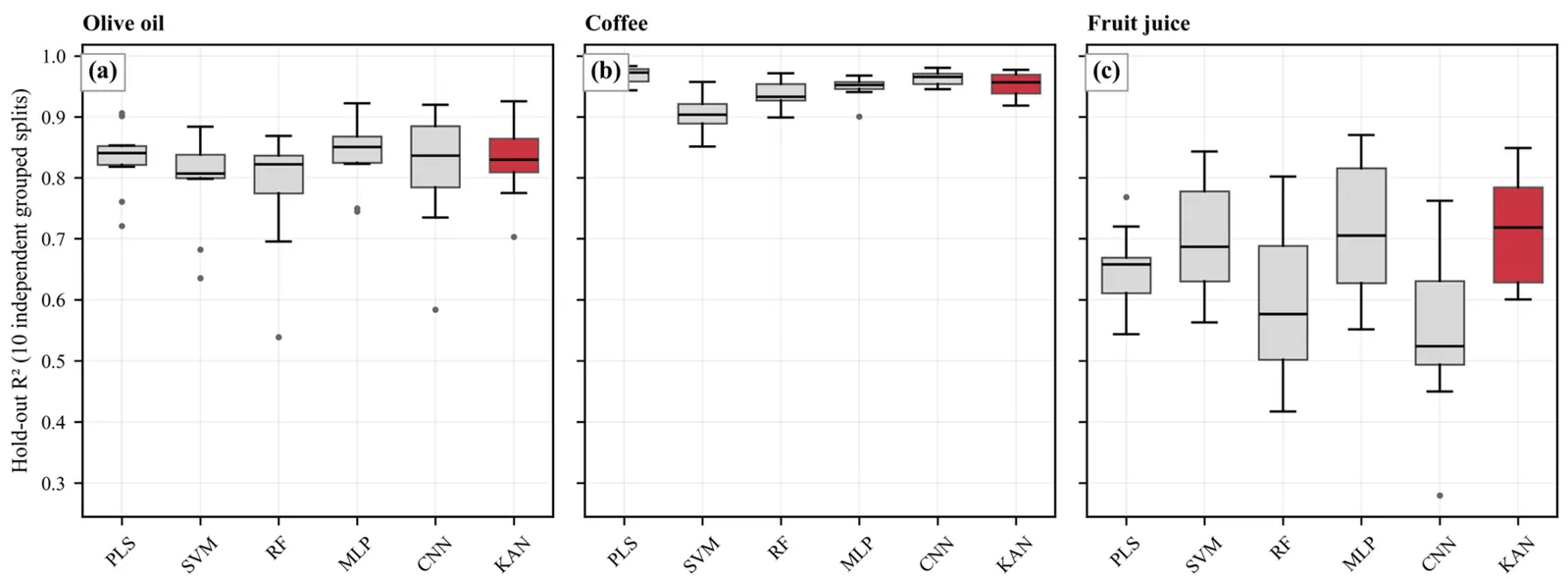
Hold-out R^2^ over ten independent grouped splits for (**a**) olive oil, (**b**) coffee, and (**c**) fruit juice.

Because the datasets are modest in size, we verified that the conclusions do not hinge on a single data split. Across ten independent grouped hold-outs, the KAN’s R^2^ was 0.83 ± 0.06 for olive oil, 0.95 ± 0.02 for coffee and 0.72 ± 0.09 for fruit juice (Figure 6). The narrow spread for olive oil and coffee confirms stable calibration; the wider spread for fruit juice is consistent with its intrinsic difficulty. Notably, the random forest and convolutional models showed much larger variance on fruit juice, reinforcing that the KAN was not only competitive on average but more reliable than the black-box learners on the hardest matrix.

### 3.5. Generalisation to Unseen Brands

The validation protocol of Section 2.5 splits by physical sample, so a model is required to generalise to blends it has not seen but may have seen other blends of the same brand. Because commercial variability across brands is a central concern for any deployed authentication method, we additionally assessed the harder case in which an entire brand is withheld.

**Figure 7:**
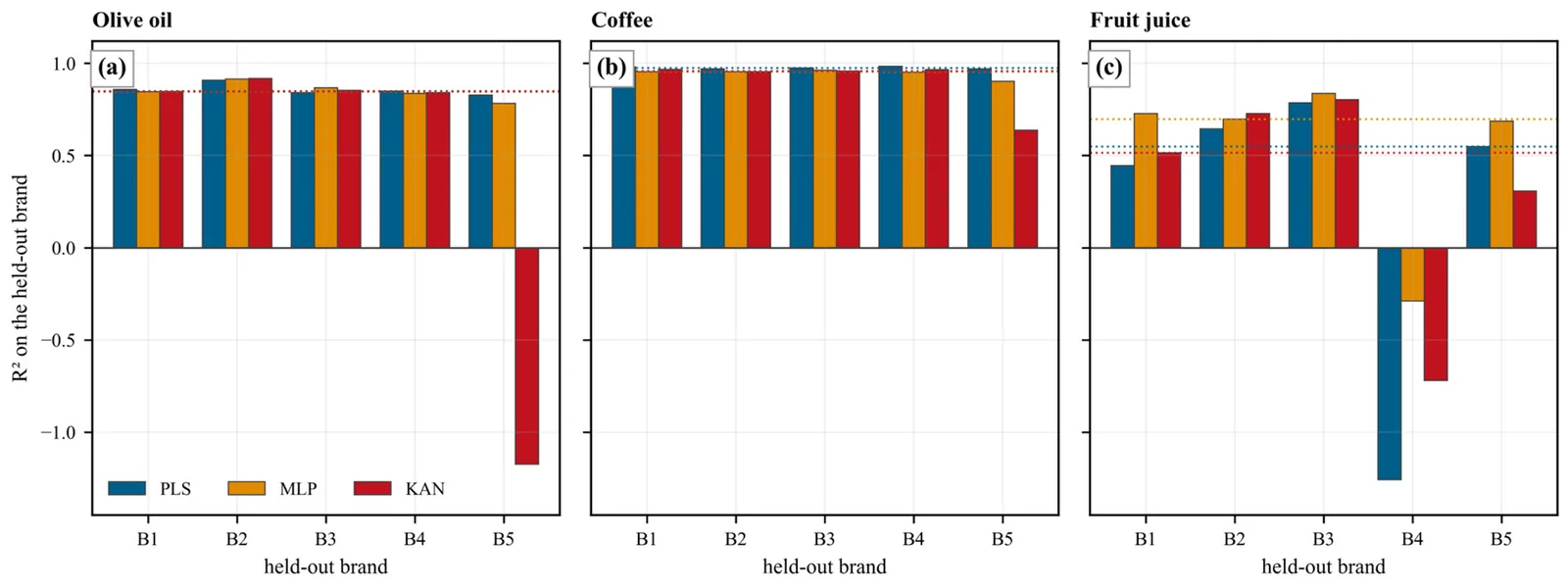
Leave-one-brand-out generalisation for (**a**) olive oil, (**b**) coffee and (**c**) fruit juice. Bars are the mean of three random seeds; dotted lines mark the per-model median across the five held-out brands.

For each matrix, models were trained on four brands and tested on the fifth, cycling through all five brands; preprocessing and the PLS projection were refitted on the four training brands in every fold. Because the KAN and the multilayer perceptron are stochastic, each fold was repeated at three random seeds (42, 7, 2024). Table 4 and Figure 7 report the per-brand results, together with the mean and the median across the five held-out brands. We report the median as well as the mean because, at five folds, a single failed fold dominates the mean.

For olive oil and coffee, the KAN transferred, as well as PLS and the multilayer perceptron, on four of the five brands (olive oil R^2^ = 0.85, 0.92, 0.85, 0.84; coffee R^2^ = 0.95–0.97), and collapsed on the fifth in both matrices. That collapse was seed-dependent—for olive oil brand 5, the three seeds gave R^2^ = 0.165, 0.325 and −4.01, and for coffee brand 5 they gave 0.83, 0.21 and 0.87—which identifies it as optimisation instability when the model is evaluated outside its training domain rather than a systematic failure to transfer. PLS, being linear and convex, showed no comparable variance (olive oil 0.857 ± 0.031; coffee 0.976 ± 0.006). For fruit juice, no model extrapolated across brands: brand 4 produced a negative R^2^ for every model at every seed (PLS −1.26; multilayer perceptron −0.20 to −0.44; KAN −0.58 to −0.83), and PLS was the weakest of the three on average, so inter-brand spectral variation in commercial orange juice evidently exceeds the adulteration signal. Most importantly, brand-level extrapolation is substantially harder than sample-grouped validation for every model tested.

We therefore restrict the scope of the paper’s claims. The calibrations reported here are validated across blends within a fixed set of five commercial brands; they are not validated across brands, and for the aqueous fruit juice matrix, brand transfer fails outright. For the KAN specifically, the run-to-run variance observed on unseen brands is a practical limitation that would need to be addressed—most obviously by ensembling over random restarts—before any deployment can be contemplated. We were not able to validate such an ensemble at the present sample size, and we prefer to report the limitation rather than a remedy we cannot yet support.

### 3.6. Ablations and a Parameter-Matched Baseline

**Figure 8:**
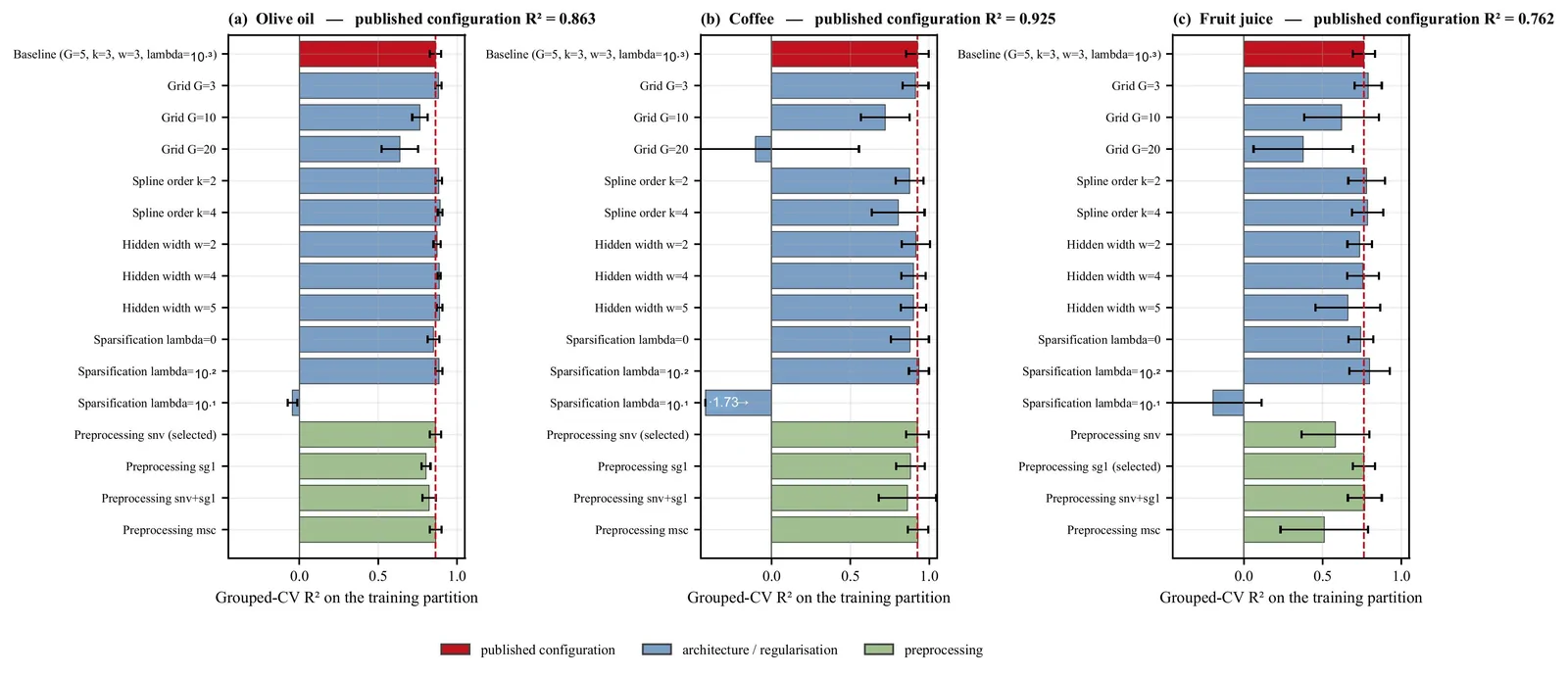
KAN ablations for (**a**) olive oil, (**b**) coffee and (**c**) fruit juice. The dashed line marks the published configuration; bars extending past the left axis are labelled with their true value. Sparsification at λ = 10^−1^ destroys the model on all three matrices.

The benchmark of Section 3.2 establishes that the KAN is competitive, but not which of its components are responsible, nor whether its behaviour is peculiar to the configuration we selected. We therefore varied each design choice in turn, holding the rest fixed at the published configuration (spline grid G = 5, spline order k = 3, hidden width w = 3, regularisation weight λ = 10^−3^). Each configuration was evaluated by stratified grouped five-fold cross-validation on the training partition, with preprocessing and the PLS projection refitted inside every fold and the per-matrix compression held at its tuned value. Because the number of configurations makes the repeated 5 × 5 protocol prohibitive, this ablation uses a single grouped five-fold; its baseline values (R^2^ = 0.863, 0.925 and 0.762) are therefore close to, but not identical with, those of Table 2 (0.855, 0.927, 0.686), and it should be read as a comparison within itself—as change relative to its own baseline—rather than against Table 2. Results are given in Table 5 and Figure 8.

Spline grid size is the most consequential architectural parameter and the only one with a monotone effect. Increasing G from 5 to 10 costs 0.10–0.20 R^2^ on every matrix, and G = 20 is catastrophic (olive oil 0.637; fruit juice 0.376; coffee −0.103, i.e., worse than predicting the mean): at 55 physical samples per matrix, a finely resolved spline basis has more degrees of freedom than the data can constrain. A coarser grid, G = 3, performed marginally better than the published G = 5 on olive oil (+0.019) and fruit juice (+0.027) and marginally worse on coffee (−0.012); we report this rather than defend G = 5, since on these data the two are equivalent within one standard deviation. Spline order had no consistent effect: k = 2 and k = 4 both slightly improved olive oil and fruit juice and degraded coffee (k = 4: −0.122), so k = 3 is a reasonable compromise rather than an optimum. Hidden width was close to irrelevant, changing the R^2^ by 0.03 at most on olive oil and coffee across widths of two, four and five; the one exception is fruit juice at w = 5 (−0.102, with the standard deviation doubling), consistent with the extra capacity overfitting the hardest matrix. This confirms that the narrow bottleneck used for symbolic extraction costs nothing in predictive accuracy.

Sparsification strength proved to be the parameter that most requires reporting. Removing the penalty entirely (λ = 0) cost 0.01–0.05 R^2^ on all three matrices, so the penalty is doing useful work. Increasing it ten-fold to λ = 10^−2^ improved every matrix (olive oil +0.022, coffee +0.010, fruit juice +0.036), so the published value is not optimal and we say so. Increasing it a further ten-fold to λ = 10^−1^ destroys the model completely (olive oil −0.045, fruit juice −0.196, and coffee −1.729, with a standard deviation of 3.17). The usable range is therefore narrow—roughly 10^−3^ to 10^−2^, with failure one decade above—and anyone reproducing this work needs that information.

The preprocessing choice confirmed each per-matrix selection made in Section 2.3 and showed how consequential the fruit juice selection is. For olive oil and coffee, SNV and MSC are statistically indistinguishable (olive oil 0.863 vs. 0.864; coffee 0.925 vs. 0.929) while the derivative filters are clearly worse, so either scatter correction would have served. For fruit juice, the choice matters a great deal: SG1 (0.762) and SNV+SG1 (0.768) far outperform SNV alone (0.581) and MSC (0.510), with the standard deviation tripling for the two non-derivative options. This is direct evidence for the argument made in Section 2.3 that derivative preprocessing is required to suppress the sloping aqueous background.

To separate the KAN’s architecture from its capacity, we matched a single-hidden-layer multilayer perceptron to the KAN’s actual trainable-parameter count on each matrix and evaluated it on the identical folds. The published KAN has 580, 1060 and 820 parameters for olive oil, coffee, and fruit juice; the matched perceptrons have hidden widths of 48, 53 and 51, giving 577, 1061 and 817 parameters. Their cross-validated R^2^ was 0.885 ± 0.008 against the KAN’s 0.863 ± 0.036 for olive oil, 0.895 ± 0.083 against 0.925 ± 0.072 for coffee, and 0.765 ± 0.114 against 0.762 ± 0.071 for fruit juice. At equal capacity, the two architectures are therefore equivalent—the perceptron marginally ahead on olive oil, the KAN marginally ahead on coffee, and indistinguishable on fruit juice, with every difference well inside one standard deviation. We accordingly withdraw parameter efficiency as an argument: the KAN’s smaller parameter count relative to the tuned baselines of Table 2 reflects those baselines having been tuned for accuracy rather than for size, not an intrinsic efficiency of the architecture. What the KAN provides that a perceptron of the same size does not is the closed-form equation, and that is the claim we retain.

Finally, we quantified what symbolic conversion costs by comparing the trained spline network with its symbolic surrogate on held-out data, averaged over the ten random seeds of Section 3.7. Olive oil: 0.840 ± 0.014 (spline) versus 0.846 ± 0.010 (symbolic). Coffee: 0.971 ± 0.008 versus 0.972 ± 0.004. Fruit juice: 0.602 ± 0.190 versus 0.666 ± 0.086. Symbolic conversion cost no accuracy on any matrix; on average, it slightly improved held-out R^2^, and on fruit juice, it also more than halved the run-to-run spread. We attribute this to the pruning-and-symbolic-fitting step acting as a strong structural regulariser—replacing free splines with a small set of analytic primitives removes precisely the flexibility that was fitting noise. The effect is within one standard deviation for olive oil and coffee, so we do not claim that symbolic conversion improves the model; the defensible conclusion is that on these data the interpretable form is obtained at no cost to accuracy.

### 3.7. Stability of the Equation and of the Explanation

**Figure 9:**
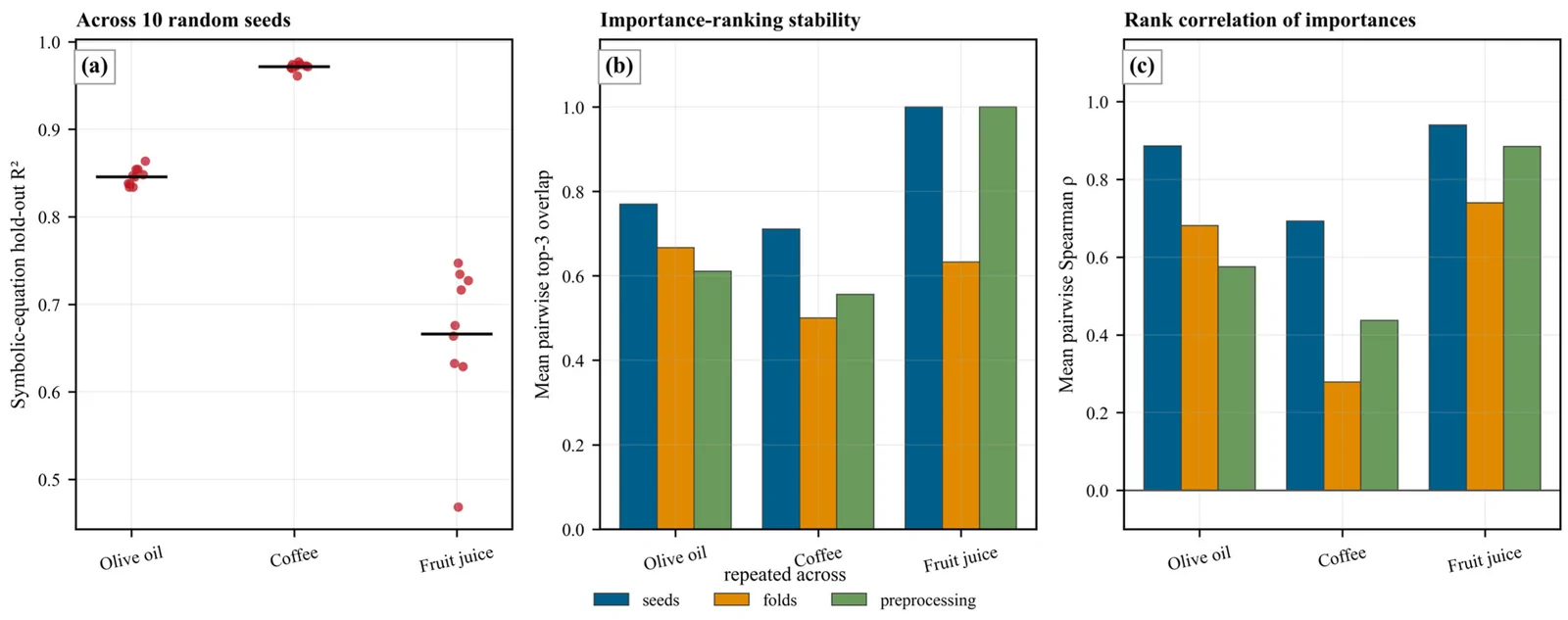
Stability of the KAN explanation. (**a**) Symbolic-equation hold-out R^2^ across ten random seeds; horizontal bars mark the mean. (**b**) Mean pairwise top-3 overlap and (**c**) mean pairwise Spearman rank correlation of the intrinsic importance vectors, repeated across seeds, cross-validation folds and preprocessing choices.

A symbolic equation is only useful if it is reproducible. We therefore repeated the whole interpretability pipeline—preprocessing, PLS projection, KAN fit, pruning and symbolic conversion—under three sources of variation: ten random seeds on the published split, the five grouped cross-validation folds, and four preprocessing choices (SNV, SG1, SNV+SG1, MSC). Every R^2^ value below is computed on data the model had not seen. The results are given in Table 6 and Figure 9.

Across ten seeds, the symbolic-equation hold-out R^2^ was 0.834–0.864 for olive oil (0.846 ± 0.010), 0.961–0.977 for coffee (0.972 ± 0.004), and 0.468–0.747 for fruit juice (0.666 ± 0.086). The olive oil and coffee distributions are tight. Fruit juice is far more variable, and we note explicitly that the value reported for that matrix elsewhere in this paper (0.47, at the fixed seed used throughout) is the minimum of its distribution rather than a typical draw; readers should take the range, not the single value, as the description of that equation’s reliability.

The mean pairwise top-three overlap of the intrinsic importance ranking was 0.77 (seeds), 0.67 (folds) and 0.61 (preprocessing) for olive oil; 0.71, 0.50 and 0.56 for coffee; and 1.00, 0.63 and 1.00 for fruit juice. The corresponding mean pairwise Spearman rank correlations were 0.89/0.68/0.58, 0.69/0.28/0.44 and 0.94/0.74/0.89. Explanations are therefore reasonably stable to random initialisation, moderately stable across training folds, and matrix-dependent across preprocessing—the last of which is expected, since a different preprocessing yields a different PLS basis and hence a genuinely different set of latent variables. Coffee is the least stable case, with a fold-to-fold rank correlation of only 0.28: which latent variables dominate depends appreciably on which blends are in the training set. Fruit juice is the most stable, because one latent variable dominates the ranking in every run. That observation carries a caution worth stating: for fruit juice, the explanation is the most reproducible of the three matrices while the prediction is the least, so ranking stability must not be read as evidence that a model is trustworthy.

Pruning retained essentially all eight latent variables in every olive oil and fruit juice run (olive oil: 10/10 for seven variables and 9/10 for the eighth; fruit juice: 9/10 for all eight). For coffee, retention varied considerably (x_1_–x_4_ and x_7_ in 10/10 runs, x_5_ in 7/10, x_8_ in 6/10, x_6_ in 3/10) and the number of retained variables ranged from five to eight. The equations are therefore compact in form—short, closed expressions over a small latent basis—but the KAN does not perform reliable variable selection on these data, and we do not claim that it does. What is reproducible is the predictive equation, not the identity of every term within it.

### 3.8. Interpretability: Equations, Chemistry and Agreement with SHAP

**Figure 10:**
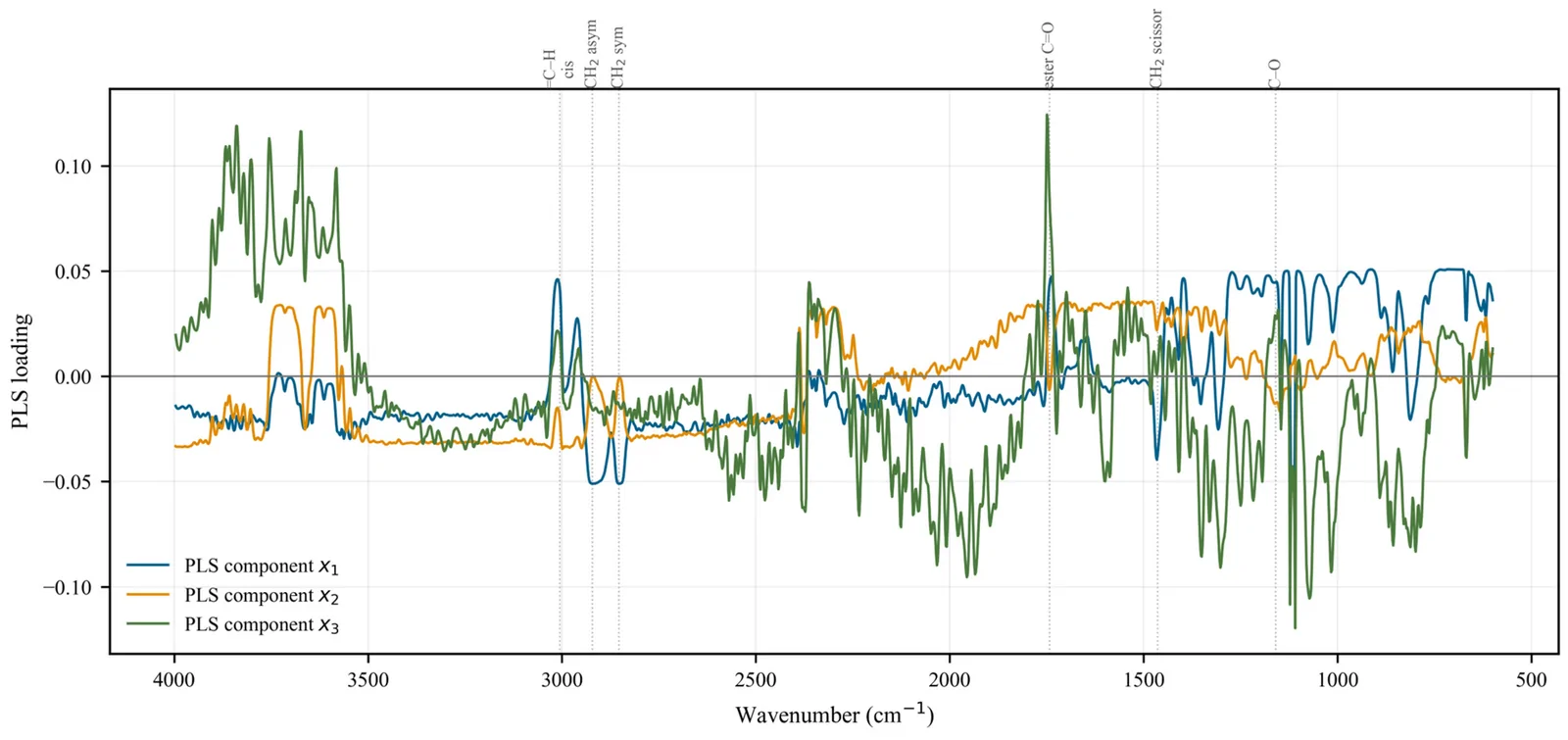
PLS component loadings of the olive oil equation’s variables overlaid on the FTIR spectrum, mapping the equation to vibrational bands.

The defining feature of the KAN is that its calibration can be written down. Pruning and symbolic conversion produced compact closed-form equations whose accuracy tracked the underlying spline network: at the fixed seed used throughout, symbolic-equation test R^2^ values were 0.83 (olive oil), 0.97 (coffee) and 0.47 (fruit juice), against spline-network values of 0.85, 0.98 and 0.69. For olive oil and coffee, the equation is therefore an honest, near-lossless surrogate for the model. The fruit juice figure requires care, and Section 3.7 gives the context: repeated over ten random seeds, symbolic conversion costs nothing on any matrix; for fruit juice, the symbolic surrogate in fact averages slightly higher than the spline network (0.666 ± 0.086 against 0.602 ± 0.190) and is markedly less variable. The single-seed value of 0.47 quoted above is the minimum of that distribution rather than a typical draw, so it should not be read as evidence that the fruit juice relationship resists symbolic compression. What it does reflect is the genuine instability of the fruit juice calibration itself, which is visible in the spline network as well. The olive oil equation (Equation (1)) combined predominantly linear terms in the leading PLS scores, with a small number of bounded non-linear terms capturing mild curvature:

(1) authentic olive-oil fraction (standardised) ≈ −1.22 x_1_ + 0.317 x_2_ − 0.398 x_3_ − 0.294 x_4_ − 0.290 x_5_ − 0.120 x_7_ − 0.150 x_8_ − 0.047 sin(3.062 x_6_ + 9.387) + 0.391 sin(1.933 x_1_ + 0.839 x_3_ + 0.524 x_7_ + 0.294 x_8_ + 0.146 e^(2.373 x^_^4^_^)^ − 0.316 sin(1.8 x_5_ − 9.996) − 8.629 − 1.593/(2.404 − 1.672 x_2_)) − 0.621

where x_1_…x_8_ are PLS scores. Overlaying the loadings of these scores on the FTIR spectrum (Figure 10) showed that the equation’s most influential variables weighted exactly the lipid regions expected to discriminate olive from sunflower oil—C–H stretching, ester C=O and fingerprint C–O bands—so the mathematics is chemically meaningful rather than an opaque fit.

**Figure 11:**
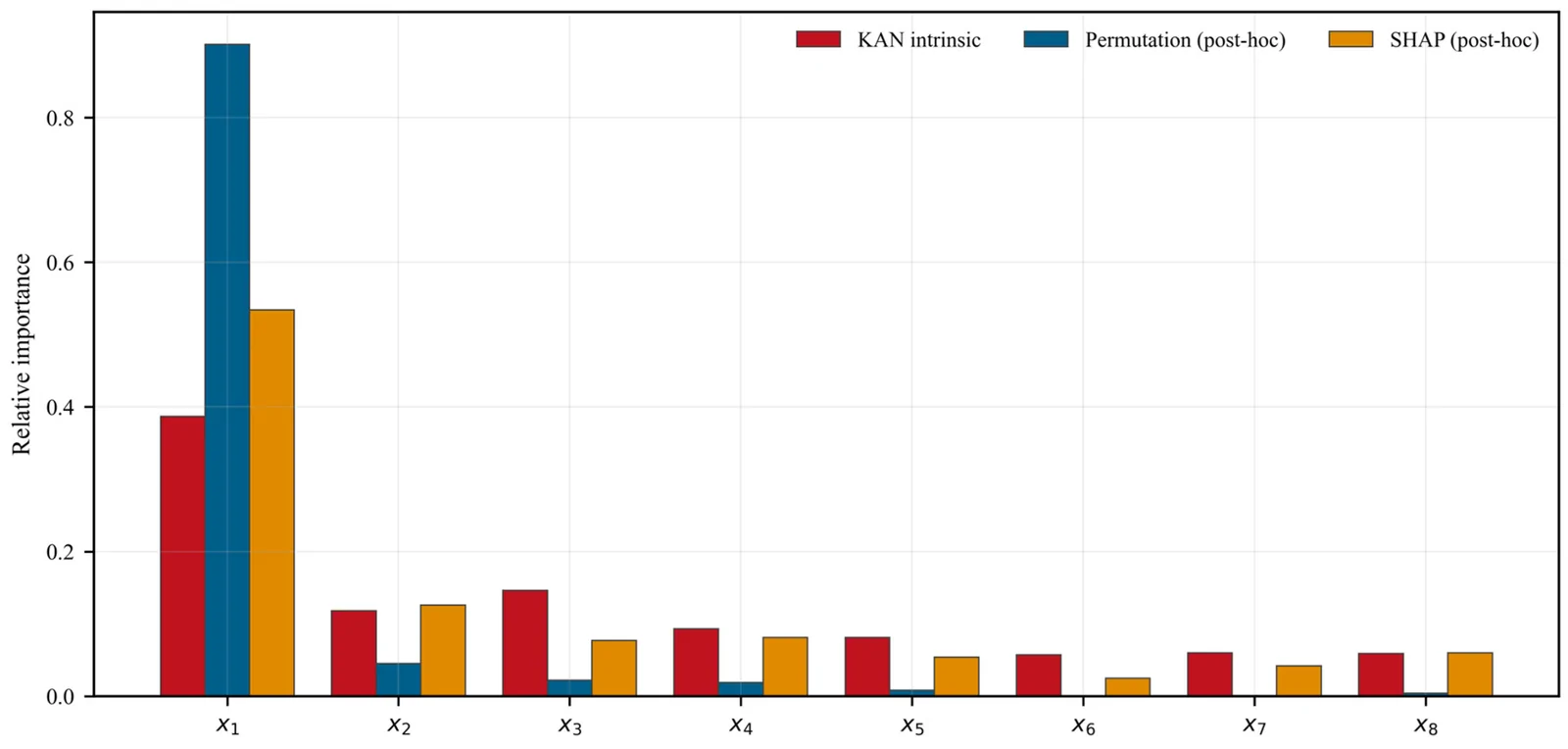
KAN intrinsic feature importance versus post hoc SHAP and permutation importance for olive oil.

To test whether this intrinsic interpretation is trustworthy, we compared the KAN’s own importance ranking with post hoc attributions obtained by permutation and by SHAP [10]. The rankings agreed closely across all three foods (Spearman ρ = 0.86 for olive oil, 0.90 for coffee and 0.88 for fruit juice against SHAP; Figure 11). In other words, the KAN intrinsically reports the same drivers that one would otherwise have to recover from a black box by an external explainer, while additionally supplying the functional form and the equation, which SHAP cannot.

### 3.9. Where the Interpretable Model Earns Its Place

Taken together, the three matrices delineate when an intrinsically interpretable non-linear model is worthwhile. Where the calibration is essentially linear (coffee), PLS is already near-optimal and the KAN’s value is on par with a transparent equation rather than offering higher accuracy. Where the calibration is subtle or non-linear (olive oil, fruit juice), the KAN matches the strongest baselines and, for fruit juice, remains more stable than the flexible black-box learners that collapse there. It is not, however, the compactness of the network that constitutes the contribution: a multilayer perceptron matched to the KAN’s parameter count performs equally well (Section 3.6), so parameter efficiency is a property of how the baselines were tuned rather than of the architecture. What distinguishes the KAN in this comparison is that it is the only model whose calibration can be written down as an equation, inspected term by term, and mapped onto vibrational bands—and, as Section 3.6 shows, that equation is obtained at no cost to accuracy. For food-authenticity analysis, where a method must be defensible to regulators and not merely accurate, the combination of competitive accuracy and intrinsic transparency—rather than any claim of uniformly superior accuracy or of greater parsimony—is the practical contribution of the approach.

### 3.10. Comparison with Previous Studies and Limitations

Our results are consistent with, and in places deliberately more conservative than, the prior spectroscopic literature, and we give specific figures rather than qualitative comparisons. For olive oil, Rohman and Che Man [20] reported R^2^ = 0.999 with a root-mean-square error of cross-validation of 0.285% for PLS quantification of palm oil in extra virgin olive oil over 1.0–50.0% (*w*/*w*) using first-derivative spectra; our grouped cross-validated calibration reaches R^2^ = 0.855 with RMSEP = 3.8%. Three differences account for the gap, and we state them rather than leave the comparison implicit: their mixture series was prepared from a single olive oil source, so between-brand variability is absent; validation was by ordinary cross-validation over the mixture series rather than by grouping replicate scans of the same blend; and palm oil differs from olive oil considerably more in its infrared fingerprint than sunflower oil does. For roasted coffee, Reis, Franca and Oliveira [23] reported correlation coefficients near 0.99 for FTIR–ATR/PLS calibrations against multiple adulterants; our leakage-free coffee calibration gives PLS R^2^ = 0.968 and KAN R^2^ = 0.927. For fruit juice, our detection limits (30.7–55.5% apple juice in orange juice) are consistent with the recognised difficulty of discriminating aqueous sugar–acid systems by vibrational spectroscopy [1,24,25], and are the reason nuclear magnetic resonance and dual-spectrometer near-infrared approaches are commonly preferred for this particular authentication problem. That FTIR/NIR chemometric adulteration analysis is well suited to a food-chemistry readership is established by in-scope precedents such as the near-infrared versus mid-infrared saffron study of Amirvaresi et al. [26]. Across these comparisons, the distinguishing feature of the present work is not higher accuracy—it is lower, and deliberately so—but the pairing of honestly validated accuracy with an intrinsic closed-form equation and a band-level interpretation that the cited approaches do not provide.

Several limitations should be borne in mind, and the additional experiments reported above have sharpened rather than softened them. The adulterated samples were laboratory-prepared binary blends rather than market-collected products, so the calibrations capture controlled two-component mixing rather than the full variability of commercial fraud. The number of independent commercial brands per matrix is five; the leave-one-brand-out results of Section 3.5 show directly that this is insufficient for brand-level generalisation, with every model degrading and none succeeding for fruit juice. The number of physical samples per food is modest (55 per matrix), which—together with the replicate structure of the spectra—is why we base our conclusions on repeated, sample-grouped cross-validation and a ten-split robustness check rather than on any single split.

The reference values used throughout are nominal mixing ratios—volumetric for the liquid matrices and gravimetric for coffee—and were not verified after preparation by an independent reference method such as gas chromatography–mass spectrometry, high-performance liquid chromatography or nuclear magnetic resonance. Any preparation error, incomplete homogenisation, or matrix interaction therefore propagates directly into the calibration targets, and the reported limits of detection are conditional on the nominal compositions being accurate. Because the symbolic equations are fitted against those same targets, the chemical interpretation inherits the same assumption. Independent compositional confirmation should be regarded as a prerequisite for any quantitative deployment claim.

Detection is screening-level. For the aqueous orange-/apple-juice system, the limit of detection is high for every model and the confidence intervals do not separate them, so this matrix is best regarded as a qualitative to semi-quantitative screening case rather than a trace-detection method.

The KAN’s advantage is not parameter efficiency. A multilayer perceptron matched to the KAN’s parameter count reaches equivalent accuracy on all three matrices (Section 3.6); therefore, the architecture’s distinguishing property is the closed-form equation it yields, not a better accuracy-per-parameter trade-off. Its symbolic equations are reproducible in predictive terms but do not identify a stable sparse variable subset (Section 3.7), and on fruit juice the equation is appreciably less reliable than on the other two matrices.

Finally, the spectra originate from a single instrument and laboratory. External validation using independently acquired spectra, additional instruments, and genuinely market-sourced adulteration remains a necessary next step before routine deployment.

## 4. Conclusions

An intrinsically interpretable Kolmogorov–Arnold network was evaluated for FTIR-based detection and quantification of adulteration in olive oil, coffee and fruit juice under strictly leakage-free, sample-grouped validation. The compact KAN was consistently competitive—statistically indistinguishable from the strongest baselines for olive oil and fruit juice, and close to the best linear model for near-linear coffee—while providing closed-form equations whose variables correspond to recognised vibrational bands and whose importance rankings agree with post hoc SHAP attribution. Converting the trained network into its symbolic form cost no accuracy on any matrix.

The boundaries of the result are equally clear and we state them together. Linear PLS remains the better model where the chemistry is linear. The multilayer perceptron was numerically strongest on fruit juice, where the symbolic equation is also the least reliable. A parameter-matched multilayer perceptron matches the KAN’s accuracy, so parsimony alone is not the contribution. The extracted equations are reproducible as predictors but do not select a stable variable subset. Under leave-one-brand-out validation, every model degraded, with the KAN additionally showing run-to-run instability on unseen brands, and no model succeeded on fruit juice.

Within these bounds, the KAN offers a combination of competitive accuracy and genuine, intrinsic transparency that is well suited to exploratory and method-development work in food-authenticity analysis, and that no other model in this comparison provides. It is not, based on this evidence, ready for routine deployment. Future work should target the three gaps this study exposes—many more independent brands and instruments, market-collected rather than laboratory-prepared adulteration, and stabilisation of KAN training under distribution shift—most plausibly by ensembling over random restarts, which our results suggest would remove the failure mode we observed, but which we could not validate at the present sample size.

## Figures and Tables

**Table 1:** Datasets and group structures.

Food	Adulterant	Range (Step)	Spectra	Samples	Preprocessing
Olive oil	Sunflower oil	0–30% (3%)	≈350	55	SNV
Coffee	Malt flour	0–50% (5%)	≈400	55	SNV
Fruit juice (orange)	Apple juice	0–50% (5%)	≈400	55	SG1

SNV = standard normal variate transformation; SG1 = first-derivative Savitzky–Golay filter.

**Table 2:** Benchmark: repeated 5 × 5 group cross-validation and independent hold-out (per food, per model). Params refers to the number of trainable parameters (left blank where not applicable).

Food	Model	CV R^2^ (Mean ± SD)	RPD	RMSEP%	F1	Params
Olive oil	PLS	0.855 ± 0.059	2.75	3.89	0.931	14,104
Olive oil	SVM	0.823 ± 0.061	2.49	4.17	0.901	
Olive oil	RF	0.833 ± 0.100	2.67	3.93	0.891	
Olive oil	MLP	0.864 ± 0.048	2.81	3.86	0.928	2881
Olive oil	CNN	0.846 ± 0.072	2.73	3.92	0.916	43,457
Olive oil	KAN	0.855 ± 0.061	2.77	3.81	0.927	580
Coffee	PLS	0.968 ± 0.021	6.11	2.61	0.988	14,104
Coffee	SVM	0.845 ± 0.105	2.78	4.51	0.803	
Coffee	RF	0.933 ± 0.046	4.25	3.36	0.945	
Coffee	MLP	0.928 ± 0.036	4.02	3.54	0.912	10,369
Coffee	CNN	0.954 ± 0.040	5.43	2.47	0.989	43,457
Coffee	KAN	0.927 ± 0.047	4.19	3.08	0.936	1060
Fruit juice	PLS	0.738 ± 0.189	2.15	10.00	0.913	14,104
Fruit juice	SVM	0.708 ± 0.240	2.05	10.00	0.895	
Fruit juice	RF	0.396 ± 0.980	1.75	9.03	0.882	
Fruit juice	MLP	0.774 ± 0.188	2.44	8.44	0.922	2881
Fruit juice	CNN	0.372 ± 0.533	1.48	9.18	0.903	43,457
Fruit juice	KAN	0.686 ± 0.373	2.14	12.48	0.891	820

**Table 3:** Limit of detection and limit of quantification (IUPAC 3σ convention) with 95% cluster-bootstrap confidence intervals, per food and model.

Food	Model	LOD (% Adulterant)	95% CI	LOQ (% Adulterant)	95% CI
Olive oil	PLS	13.8	10.3–18.3	41.7	31.3–55.6
Olive oil	SVR	14.9	11.9–18.7	45.1	35.9–56.6
Olive oil	RF	14.3	10.4–20.0	43.2	31.4–60.6
Olive oil	MLP	13.8	10.2–18.2	41.8	30.9–55.1
Olive oil	CNN	14.2	11.3–18.8	43.0	34.2–57.1
Olive oil	KAN	13.7	9.8–19.0	41.5	29.8–57.6
Coffee	PLS	8.7	6.5–11.3	26.5	19.8–34.2
Coffee	SVR	14.9	12.3–16.5	45.2	37.2–50.1
Coffee	RF	11.4	8.1–14.6	34.4	24.4–44.1
Coffee	MLP	11.8	8.8–14.2	35.7	26.6–43.1
Coffee	CNN	8.2	5.9–10.5	24.9	17.9–31.8
Coffee	KAN	10.3	7.4–12.6	31.1	22.4–38.3
Fruit juice	PLS	38.3	24.4–44.7	115.9	74.0–135.4
Fruit juice	SVR	37.1	25.4–41.9	112.4	77.1–127.1
Fruit juice	RF	34.2	25.9–44.5	103.6	78.4–134.7
Fruit juice	MLP	30.7	23.0–35.1	93.1	69.8–106.4
Fruit juice	CNN	34.5	26.0–53.2	104.6	78.8–161.2
Fruit juice	KAN	55.5	22.7–79.7	168.3	68.9–241.6

**Table 4:** Leave-one-brand-out generalisation: R^2^ on the held-out brand, averaged over three random seeds per fold.

Food	Model	LOBO R^2^ (Mean ± SD)	Median	Worst Brand
Olive oil	PLS	0.857 ± 0.031	0.849	0.827
Olive oil	MLP	0.850 ± 0.048	0.845	0.783
Olive oil	KAN	0.457 ± 0.912	0.846	−1.174
Coffee	PLS	0.976 ± 0.006	0.975	0.970
Coffee	MLP	0.945 ± 0.024	0.954	0.902
Coffee	KAN	0.896 ± 0.145	0.957	0.636
Fruit juice	PLS	0.233 ± 0.843	0.548	−1.259
Fruit juice	MLP	0.532 ± 0.463	0.697	−0.289
Fruit juice	KAN	0.327 ± 0.616	0.514	−0.719

**Table 5:** KAN ablations: grouped five-fold cross-validated R^2^ on the training partition, with each configuration differing from the published one in a single respect.

Food	Ablation	CV R^2^ (Mean ± SD)	Δ vs. Baseline
Olive oil	Baseline (G = 5, k = 3, w = 3, λ = 1 × 10^−3^)	0.863 ± 0.036	—
Olive oil	Grid G = 3	0.882 ± 0.019	+0.019
Olive oil	Grid G = 10	0.764 ± 0.049	−0.099
Olive oil	Grid G = 20	0.637 ± 0.117	−0.226
Olive oil	Spline order k = 2	0.884 ± 0.021	+0.021
Olive oil	Spline order k = 4	0.892 ± 0.014	+0.029
Olive oil	Hidden width w = 2	0.873 ± 0.023	+0.010
Olive oil	Hidden width w = 4	0.887 ± 0.010	+0.024
Olive oil	Hidden width w = 5	0.889 ± 0.017	+0.026
Olive oil	Sparsification λ = 0	0.850 ± 0.036	−0.013
Olive oil	Sparsification λ = 1 × 10^−2^	0.885 ± 0.022	+0.022
Olive oil	Sparsification λ = 1 × 10^−1^	−0.045 ± 0.030	−0.908
Olive oil	Preprocessing snv (selected)	0.863 ± 0.036	−0.000
Olive oil	Preprocessing sg1	0.803 ± 0.028	−0.060
Olive oil	Preprocessing snv+sg1	0.822 ± 0.042	−0.041
Olive oil	Preprocessing msc	0.864 ± 0.037	+0.001
Coffee	Baseline (G = 5, k = 3, w = 3, λ = 1 × 10^−3^)	0.925 ± 0.072	—
Coffee	Grid G = 3	0.913 ± 0.082	−0.012
Coffee	Grid G = 10	0.720 ± 0.154	−0.205
Coffee	Grid G = 20	−0.103 ± 0.657	−1.028
Coffee	Spline order k = 2	0.875 ± 0.089	−0.050
Coffee	Spline order k = 4	0.803 ± 0.167	−0.122
Coffee	Hidden width w = 2	0.915 ± 0.089	−0.010
Coffee	Hidden width w = 4	0.900 ± 0.078	−0.025
Coffee	Hidden width w = 5	0.900 ± 0.080	−0.025
Coffee	Sparsification λ = 0	0.878 ± 0.120	−0.047
Coffee	Sparsification λ = 1 × 10^−2^	0.934 ± 0.064	+0.009
Coffee	Sparsification λ = 1 × 10^−1^	−1.729 ± 3.169	−2.654
Coffee	Preprocessing snv (selected)	0.925 ± 0.072	−0.000
Coffee	Preprocessing sg1	0.881 ± 0.091	−0.044
Coffee	Preprocessing snv+sg1	0.862 ± 0.181	−0.064
Coffee	Preprocessing msc	0.929 ± 0.065	+0.004
Fruit juice	Baseline (G = 5, k = 3, w = 3, λ = 1 × 10^−3^)	0.762 ± 0.070	—
Fruit juice	Grid G = 3	0.789 ± 0.086	+0.027
Fruit juice	Grid G = 10	0.620 ± 0.238	−0.142
Fruit juice	Grid G = 20	0.376 ± 0.315	−0.386
Fruit juice	Spline order k = 2	0.779 ± 0.116	+0.017
Fruit juice	Spline order k = 4	0.785 ± 0.099	+0.023
Fruit juice	Hidden width w = 2	0.735 ± 0.078	−0.027
Fruit juice	Hidden width w = 4	0.755 ± 0.101	−0.006
Fruit juice	Hidden width w = 5	0.660 ± 0.206	−0.102
Fruit juice	Sparsification λ = 0	0.742 ± 0.079	−0.020
Fruit juice	Sparsification λ = 1 × 10^−2^	0.798 ± 0.129	+0.036
Fruit juice	Sparsification λ = 1 × 10^−1^	−0.196 ± 0.309	−0.958
Fruit juice	Preprocessing snv	0.581 ± 0.216	−0.181
Fruit juice	Preprocessing sg1 (selected)	0.762 ± 0.070	+0.000
Fruit juice	Preprocessing snv+sg1	0.767 ± 0.107	+0.006
Fruit juice	Preprocessing msc	0.510 ± 0.278	−0.252

**Table 6:** Stability of the KAN explanation: mean pairwise agreement of the intrinsic importance ranking across ten random seeds, five grouped cross-validation folds, and four preprocessing choices.

Food	Top-3 Overlap (Seeds/Folds/Preproc.)	Spearman ρ (Seeds/Folds/Preproc.)	Symbolic Hold-Out R^2^ (Mean ± SD)	Range Over Seeds
Olive oil	0.77/0.67/0.61	0.89/0.68/0.57	0.846 ± 0.010	0.834–0.864
Coffee	0.71/0.50/0.56	0.69/0.28/0.44	0.972 ± 0.004	0.961–0.977
Fruit juice	1.00/0.63/1.00	0.94/0.74/0.89	0.666 ± 0.086	0.468–0.747

## Data Availability

The data presented in this study are openly available in kan-ftir-food-adulteration at https://github.com/abdulhamidbatayhi123/kan-ftir-food-adulteration (accessed on 19 August 2026).
